# Can trade and security alliance help reduce interstate war?

**DOI:** 10.1371/journal.pone.0304482

**Published:** 2024-06-20

**Authors:** Seung-Whan Choi

**Affiliations:** Department of Political Science, University of Illinois, Chicago, Illinois, United States of America; KAIST: Korea Advanced Institute of Science and Technology, REPUBLIC OF KOREA

## Abstract

This study explains how the gap between theory and empirical research hinders scientific progress in the area of international political economy. To demonstrate this point, I use Chen’s Extended Dependence Theory, which challenges liberal peace theory but fails to provide supporting empirical evidence. Chen contends that it is not trade dependence between two states that fosters peace but a challenger’s trade relations with the defense-pact partners of the target. Although Chen criticizes liberal peace proponents whose primary concern is how to deter war, his empirical analysis is confined to how to decrease (fatal) militarized disputes short of war. I argue that for his theory to succeed, it must be validated against the most severe and intense form of conflict. Using statistical tests and substantive significance, I uncover no peace-building effect, with regards to war, attributable to Extended Dependence. It appears that the Extended Dependence variable exhibits a ceiling effect. Future research should explain why economic ties and security institutions fail to work together to lower the risk of the most destructive form of conflict.

## Introduction

Liberal peace theory postulates that “economic development and mutual trade are powerful inhibitors of *war*” [1 page 292, emphasis added; see also 2,3]. In empirical studies, mutual trade is usually referred to as dyadic trade dependence. It is measured as the sum of imports and exports between the challenger and target states divided by the challenger’s GDP [see also 4]. Employing this measurement, many empirical studies show that “bilateral trade flows reduce the probability of a bilateral *war*” [5 page 2, emphasis added], while some provide counterexamples [e.g., 6–12].

Chen’s [13] study is one of the latest challenges, arguing that liberal peace is created not by dyadic dependence (the challenger’s trade dependence on the target state) but by Extended Dependence (the challenger’s trade dependence on the military allies of the target state, measured as the sum of the challenger’s trade volume with the target’s allies, divided by the challenger’s GDP). Chen contends that states shy away from aggression if they fear trade retaliation from the target’s security allies. More specifically, aggression is demotivated when security allies are likely to reduce trade with the challenger, disturb trade relations of the challenger with economic sanctions, and/or undermine the challenger’s alternative market choices. Chen’s analysis for the years 1951–2010 shows strong and consistent evidence for the pacifying effect of Extended Dependence on all and fatal MIDs. Chen concludes that his Extended Dependence Theory prevails over the conventional trade dependence theory.

Chen’s work contributes to the ongoing debate on how to create international peace and draws serious scholarly attention by demonstrating why economic and security linkages matter. As of March 14, 2024, Chen’s work already collects thirteen citations, though it was published only three years ago. Considering King’s [14 page 445] lament that the “modal number of citations to articles in political science is zero,” Chen’s total citation count indicates that his theory and empirical findings have been well-received among peers–an early, favorable sign for becoming an authoritative study in the field of international relations. Yet, what would happen if we put Chen’s work under the microscope? Can Chen’s work live up to its scholarly recognition? To the best of my knowledge, a study has challenged Chen’s conclusion. Feldman and Shipton [15 page 857] discover that “the ability of trade to deter conflict declines when states possess substantial maritime capabilities.” Feldman and Shipton’s finding warrants further validity testing of Chen’s Extended Dependence theory.

My argument is that Chen neglects to address the causes of war, the primary concern of liberal peace researchers. Instead, he focuses solely on low-intensity conflict. In his theoretical discussion, Chen criticizes liberal peace studies and asserts that Extended Dependence outperforms dyadic dependence in deterring war and international conflict. Yet, Chen confines his empirical analysis to low-intensity conflict–(fatal) militarized disputes. Chen does not explain why he omits interstate war in the empirical testing despite the fact that studying war is the most important topic for peace researchers. As is well-known, war is rare, but it can cause immense damage to human life, living conditions, economic endeavors, goods and services, and the environment [16].

If India and Pakistan engaged in a full-scale nuclear war, it would engender devastating outcomes. Accordingly, understanding the causes of war is at the heart of liberal peace literature. For example, when Oneal and Russett [17] show the peace-building effect of trade dependence, they test all three levels of conflict–all MIDs, fatal MIDs, and war. Compared to the gravity of war’s destructive consequences, many low-intensity militarized disputes can happen by accident or human errors, irrespective of Extended Dependence [18,19]. Chen should have conducted a comprehensive examination in which the effects of extended and trade dependence are evaluated through a side-by-side comparison of each level of conflict (all MIDs, fatal MIDs, and war). Otherwise, Chen’s claim that Extended Dependence outweighs trade dependence is not warranted because he did not examine how to fend off the most destructive political violence, overstretching the implications of his empirical findings (based solely on all and fatal MIDs).

In this study, I empirically address what Chen’s study is lacking–the impact of Extended Dependence on interstate *war*. Chen’s theory states that during the deliberation of conflict initiation, a challenging state must assess the possible outcomes and consequences of its military actions. One of the most crucial assessments to incorporate is how to deal with the target’s military allies that would unfavorably react to the challenging state. The rationale is that “among all the defense pacts that are still effective or that terminated after 1950, approximately 73% of the treaties require *unconditional obligations*, regardless of who the challenger is” [13 page 249, emphasis added). The challenger has fewer incentives to attack the target if it wishes to avoid trade retaliation from the target’s security allies. This reasoning must also be valid in case of the outbreak of war–the most destructive aggression warrants devastating retaliatory action.

My reasoning is confirmed by Chen’s empirical findings, which discuss the relative importance of all MIDs and fatal MIDs as follows:

Relatively, the magnitude of the effect [of Extended Dependence] on reducing Fatal MIDs is larger than that on decreasing All MIDs. A plausible explanation for this finding is that defensive alliance obligations are clearer when military conflict is *more severe and intense*, where Extended Dependence can thus produce an even stronger effect on reducing the incidence of military conflict initiation (p. 253, emphasis added).

This empirical comparison suggests that retaliatory actions from the military allies of the target are more likely if the military attacks initiated by the challenging state are more severe and intense. Provided that this empirical comparison is accurate, war is more likely to serve as a tipping point for the security allies of the target to launch a trade or hot war than any low-intensity militarized disputes.

However, I contend that Chen’s theory has little to no explanatory power regarding interstate war. On paper, the security allies of the target should engage in retaliatory trade policies against the challenging state; in practice, they are lukewarm to security commitments primarily due to their economic stakes in the challenging state. To clarify my point, I introduce the example of the Falklands War, an armed conflict between Argentina and the U.K. in 1982 over two British-dependent territories in the South Atlantic. The War began on April 2, when Argentina invaded and occupied the Falkland Islands, followed by the invasion of South Georgia the next day [20,21]. Before these attacks, Argentina did not worry much about retaliatory trade action by the military allies of the U.K. Indeed, the War did not cause the British allies, such as the U.S., Canada, and Australia, to strain or sever economic relations with Argentina. Not surprisingly, Argentina’s net trade in goods did not decrease after the War, as it was $712 million in 1981, $2,764 million in 1982, and $3,716 million in 1983, according to the 2018 World Development Indicators.

Another example is the Iraqi invasion of Kuwait that started on August 2, 1990. At first glance, the seven-month-long Iraqi military occupation of Kuwait may appear to be in line with Chen’s Extended Dependence Theory: the Iraqi invasion was not deterred because Kuwait did not have major military allies to fall back on. However, the Iraqi invasion led to a direct military response by a United Nations-authorized coalition of forces led by the United States, although Kuwait was not part of any significant military alliances. This was because the Iraqi invasion was severe and intense enough for 35 coalition members to take up arms against the aggressor. The Iraqi invasion also caused members of the UN Security Council to impose a near-total financial and trade embargo on the aggressor [22].

Chen’s theory also fails to explain why low-intensity militarized disputes occur between two states even when they are connected through Extended Dependence. For example, although a series of territorial disputes over the Tokdo Islands occurred between South Korea and Japan throughout history, South Korea’s military allies did not retaliate against Japan. Since the territorial disputes over a group of small islets in the East Sea that South Korea controls and Japan contests were militarized disputes without fatalities, this historical example provides a good validity test for Chen’s assertion that his theory garners strong empirical support even for minor disputes with no death toll (see Chen’s Models 1 to 4 in Table 1 on p. 254). South Korea deployed fighter jets several times to warn away Japanese planes approaching the disputed Islands and increased their patrols around the islands in response to the Japanese military presence [23]. Although Japan provoked numerous militarized disputes, the military allies of South Korea did not attempt to “(1) reduce trade with [Japan] as a consequence of direct military intervention, (2) punish [Japan] by imposing economic sanctions or implementing unfavorable trade policies, or (3) undermine [Japan’s] ability to access alternative markets to its lost trade” [13 page 2].

**Table 1:** Extended dependence and international conflict initiation.

	DV: All MIDs	DV: Fatal MIDs	DV: War
	Replicated	Replicated	
	Model 1	Model 2	Model 3
Extended Dependence	-0.009**	-0.019*	-0.072
	(0.004)	(0.008)	(0.045)
Dyadic Dependence	-0.007	0.000	-0.547
	(0.007)	(0.014)	(0.430)
Challenger Total Capability Share	0.723***	0.514	0.830*
	(0.139)	(0.300)	(0.369)
Mutual Defensive Allies Count	0.005	-0.023	0.012
	(0.011)	(0.022)	(0.039)
Dyadic Defense Pact	-0.130	-0.101	0.007
	(0.195)	(0.325)	(0.687)
Target Major Power	1.336***	0.818**	1.720***
	(0.168)	(0.286)	(0.490)
Challenger Democracy	-0.246***	-0.375***	0.209
	(0.059)	(0.106)	(0.137)
Target Democracy	-0.085	-0.087	-0.842**
	(0.069)	(0.133)	(0.309)
Contiguity	3.712***	4.589***	2.630***
	(0.166)	(0.296)	(0.400)
Distance	-0.344***	-0.440***	-0.487***
	(0.054)	(0.079)	(0.128)
Year Fixed Effects	Yes	Yes	Yes
Temporal Dependence	Yes	Yes	Yes
Observations	1,378,861	1,362,097	1,362,097

*Note*: Robust standard errors in parentheses.

*p < .05

**p < .01

***p < .001, two-tailed tests.

On the contrary, South Korea’s military allies expanded trade with Japan amid territorial disputes over the islands. For example, U.S. trade with Japan has increased, as shown in S1 Appendix. In the meantime, the U.S. has refused to curb Japanese aggression over decades. Roehrig [24 page 223] aptly assesses Washington’s position: “the U.S. government does not take a position on the sovereignty of the islands; this is a dispute that Seoul and Tokyo must resolve diplomatically, and the United States will accept whatever decision is reached between its two allies.” Roehrig’s assessment aligns with the statement of Secretary of State John Kerry at a press conference:

While the United States obviously has a strong interest in the relationship and in the security component of the relationship, it’s up to Japan and the Republic of Korea to put history behind them and move the relationship forward. And it is critical at the same time that we maintain robust trilateral cooperation, particularly in the face of North Korea’s nuclear threat [25].

It is apparent that the U.S. has no intention to honor the terms of the alliance treaty with South Korea due to its special security interests in fostering “robust trilateral cooperation,” let alone its economic stakes in Japan.

Fatal militarized interstate disputes exhibit patterns similar to militarized disputes with no fatality. For instance, the Evros River incident of December 1986 was a deadly military clash between Turkish and Greek soldiers along the Evros River near the town of Feres on the Greco-Turkish border. A Turkish soldier fatally shot a Greek soldier when they ran into each other along the River border area. Turkish and Greek soldiers continued to exchange fire for the next two hours, injuring a Greek soldier and killing two Turkish soldiers [26,27]. Though multiple fatalities were involved, these militarized disputes did not prompt the military allies of Greece to initiate any retaliatory trade war against the aggressor.

The above historical examples suggest that in the instance of limited military confrontations, such as (fatal) militarized disputes, the security allies of the target are unlikely to fulfill their unconditional obligations to defend their friends. If the security allies of the target need to live up to their promises, it must be the case that their friends are under full-scale military assaults such as interstate war. As captured in the Iraqi invasion of Kuwait, it is reasonable to expect that for the Extended Dependence Theory to be true, empirical evidence must be shown for the pacifying effect of Extended Dependence on war, which is the most severe and intense form of conflict and thus the one that security allies of the target are likely to act on. The causal connection among trade, alliance, and war must be the crux of the policy significance of Chen’s [13] contribution. Yet, as noted, his empirical analysis neglects to empirically test the pacifying effect of Extended Dependence on interstate war despite its great importance in the liberal peace literature.

When I reanalyze Chen’s study, taking the interstate war into account, I discover no pacifying effect attributable to Extended Dependence. This null finding is also verified by robustness tests such as a Heckman selection model and a generalized method of moments model to control for endogeneity bias. This null finding should be noted as an effort to inseminate scientific truth, as Laitin [28 page 5] points out that “we need to incentivize the publication of replications and null results.” Recently, Alrababa’h et al. [29] also underscore the importance of null findings in pursuing scientific discovery.

Chen’s Extended Dependence variable appears to be subject to a ceiling effect. States are unlikely to fulfill their alliance commitments when they anticipate that their retaliatory trade action will leave them worse off than the status quo. Initiating war may be too costly for the allies; their overriding interest is fostering and protecting economic prosperity. Conversely, when the rewards are high enough to offset the costs–stakes are too high for the allies not to enter the war (e.g., disruption of oil production, control of trade routes, etc.)–states may come to the help of another state even in the absence of military alliance, as seen in the example of the first Persian Gulf War.

It is worth reemphasizing that if military allies have trade interests with the challenger, they have enormous incentives to stand aside to preserve the benefits of the economic relationship with the challenger rather than punishing it for its aggression. To protect their economic interests, the military allies may either break their alliance commitment with the target or find a way to argue that the terms of the alliance treaty do not apply under some special circumstances [30]. In addition, military allies of target states are not cohesive units. Some military allies may calculate one way and others differently. These mixed signals diverge the aggressor’s calculations, so the significant impact of Extended Dependence on interstate war is unlikely to materialize.

Another interpretation of the null finding is that the military allies of the target consider both (a) the salience of the conflict and (b) the costs of (military and economic) intervention. In the case of low-intensity conflict, the salience and the costs are low. In the event of war, both are high. For fatal militarized disputes, the salience is much higher than for low-intensity disputes (given the obligations) but still lower than for war, and the costs are higher than for low-intensity disputes but much lower than for war. Therefore, the allies’ decision involves a tradeoff. The effect of Extended Dependence is thus higher for fatal MIDs but attenuates when it comes to war and low-severity disputes.

Overall, the null findings of my reexamination align with several other existing studies in related areas. For example, Kim [11] finds evidence that economic linkages are associated with militarized disputes but do not matter in reducing the risk of war.

## Replication and extended empirical analysis

In this section, I explain how to operationalize interstate war, replicate the main models of Chen’s study, and present my extended empirical analysis along with a series of robustness tests.

Chen’s study builds a statistical model as follows:

(Fatal) Militarized Interstate Disputes_*ijt*_ = f (Extended Dependence_*ijt-1*_, Dyadic Dependence_*ijt-1*_, Control Variables_*ijt-1*_)

Since the dependent variable is rare and dichotomous, the study chooses a rare event logistic regression model as an estimation method. All independent variables are lagged one year to avoid potential endogeneity and reverse causality. The statistical model accounts for time dependence with the cubic polynomial approximation: *t*, *t*^*2*^, and *t*^*3*^. In addition, to control for aggregate temporal trends, the model includes year fixed-effects.

For my reexamination, I modify Chen’s model specification by employing the dependent variable of interstate war in place of (fatal) militarized disputes. To construct the war variable, I rely on the same data source and operationalization that Chen’s study specifies. Chen’s study collects (fatal) militarized disputes from Maoz et al.’s [31] dyadic militarized disputes dataset version 3.1, so I use the dyadic war collection to operationalize my interstate war variable. Consistent with liberal peace studies [e.g., 17], I create a dichotomous variable for the initiation of an interstate war that caused at least 1000 battle deaths. The variable is coded as “1” when State A in the directed dyad initiates a new war against State B in a given year and “0” otherwise. As in Chen’s study, I exclude joiners. Consistent with Chen’s operationalization of all and fatal MIDs, I remove the years in which a pair of states are engaged in an ongoing war. This is a common practice in peace studies to avoid temporal dependence.

In his study, Chen [13 page 20] draws Table 1 to introduce the baseline models. Models 4 and 8 perform the most comprehensive test on the Extended Dependence Theory. Chen demonstrates that Extended Dependence is a strong and positive determinant of peace in competition with dyadic dependence and other control variables. The dependent variable in the models is all militarized disputes (Model 4) and fatal militarized disputes (Model 8). For my extended analysis, I choose to replicate Models 4 and 8 since they are the most inclusive models. In doing so, I test the effect of Extended Dependence on interstate war. Note that I choose the same significance levels used in Chen’s study: 0.05, 0.01, and 0.001

Table 1 displays my reanalysis. Model 1 shows the replicated estimates of Chen’s Model 4; Model 2 indicates the replicated results of Chen’s Model 8; and Model 3 represents the extended results when the dependent variable is interstate war. Using the replication materials posted on Dataverse, I successfully reproduce Chen’s Models 4 and 8 without any difficulty. The magnitude and sign of the coefficients are the same as Chen’s reported findings. The replicated results show that Extended Dependence matters in reducing both levels of conflict. Chen’s study emphasizes that since “defensive alliance obligations are clearer when military conflict is more severe and intense” (p. 21), the pacifying effect of Extended Dependence on fatal militarized disputes appears to be stronger than that of all militarized disputes.

Chen’s emphasis is consistent with my line of reasoning–whether the peace-building effect of Extended Dependence materializes is dependent on the severity and intensity of the conflict. In other words, for Chen’s theory to be accurate, a stronger and negative effect of Extended Dependence on interstate war must also emerge since war is far “more severe and intense” than any other type of conflict. However, my reanalysis shown in Model 3 deviates considerably from the expectation. The Extended Dependence variable is not significantly different from zero; the allied friends of the target state are neither more nor less likely to launch a (trade) war against the aggressor.

**Fig 1:**
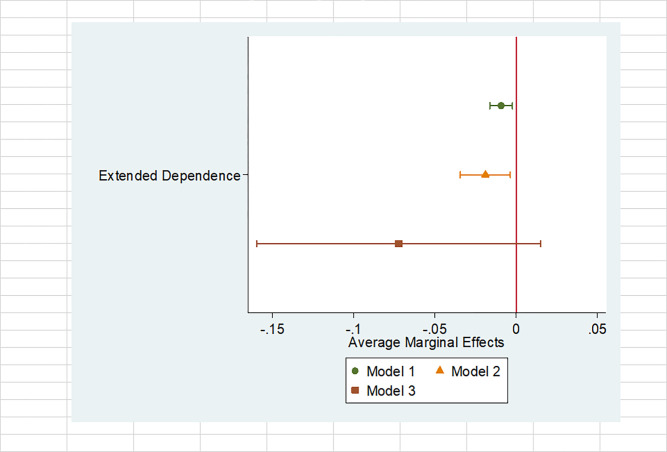
Average marginal effects.

Although the null finding comes from the same significant tests that Chen employs in his article, it is interesting to further investigate how close the extended dependence theory comes to being supported in Model 3. Put another way, I tabulate regression coefficients and standard errors since this has long been a standard way of communicating results with readers. However, significance tests may not be meaningful in a practical sense when the sample size increases [32]. For further empirical verification, I discuss the substantive effect of Extended Dependence using a graphical presentation. I plot average marginal effects in Fig 1. based on Table 1. Average marginal effects give us an effect on the probability of conflict, ranging from 0 to 1. They represent the average change in the probability when each variable increases by one unit. The figure indicates no meaningful relation between Extended Dependence and War. This visual pattern confirms that the extended dependence theory is ineffective in explaining the war outbreak.

Both significance and substantive tests indicate that while the Extended Dependence variable works out for all and fatal MIDs, it is not a peacebuilding force in case of interstate war. What do these findings mean for students of liberal peace? Overall, it is good news that at least two of the three most frequently discussed types of conflict in the literature may be reduced as Extended Dependence increases. However, it is also bad news, as it may not be strong enough to prevent war.

In his Online Appendix, Chen provides an extensive sensitivity analysis of the main findings of the first table reported on page 20. His study offers nine robustness checks in the online supplementary material (see pp. 8–21). The pacifying effect of Extended Dependence is confirmed through each of these robust tests. I choose to replicate all the robustness tests except for one that is not reproducible due to a lack of information on whether the challenging state was a revisionist in a dyadic war. Maoz et al.’s [31] directed dyadic war dataset used for my empirical analysis classifies the challenging states into only two categories: primary initiators and targets.

S2 Appendix shows my re-estimates of Chen’s sensitivity analysis. The estimated results are obtained after I introduce a sole modification to his initial model specification, namely the dependent variable of interstate war. Similar to the results in Table 1, I do not see the war-reducing effect of the Extended Dependence variable across the models except for one where the *p*-value is 0.04. Below, I discuss each sensitivity test in detail.

Model 1 limits the dyadic observations to politically relevant dyads. Politically relevant dyads involve either a major power in a dyad or border sharing between two states in a dyad. As Weede [33 page 396] points out, “only in this relatively small subset of dyads is there a possibility for irreconcilable conflicts of interest to arise and create a substantial risk of war.” Given the increased probability of conflict among politically relevant dyads, I expect to see a pacifying effect of Extended Dependence on war. Yet, as shown in Model 1, Extended Dependence does not achieve significance. By summing Extended Dependence and Dyadic Dependence, Model 2 combines the two terms. Chen creates the new measure to capture the combined effect of the two terms as they are similar indicators for trade benefits. Although Chen’s study reports a significant influence of the combined term on (fatal) militarized disputes, my extended model fails to find the same effect on war.

Model 3 adds a control variable that captures whether the potential target is a defensive ally of the U.S. The added variable, Target U.S. Defense Pact, fails to achieve significance, as does the Extended Dependence variable. Model 4 adds a control variable for whether the potential target is a member of NATO. The added variable to this model, Target NATO Member, is not significantly different from zero, and again, the Extended Dependence variable does not emerge as a significant predictor of interstate war. Model 5 considers three additional factors pertaining to third-party alliance relationships: (1) the total number of the target’s defensive allies; (2) alliance portfolio similarity between the challenger and the target; and (3) the number of co-IGO memberships. Among these three controls, the Alliance Portfolio Similarity variable alone achieves significance. But in the presence of the three additional factors, the Extended Dependence variable remains insignificant.

Chen’s Online Appendix defines outlying observations of Extended Dependence when their value is greater than 200% (p. 11). Model 6 shows re-estimated results after removing those outliers and changing the dependent variable to interstate war. The pacifying effect of Extended Dependence does not materialize. The results in Model 7 are obtained after implementing listwise deletion for Dyadic Dependence. Listwise deletion aims to remove the missing observations in the original data of Dyadic Dependence in stead of filling them with zeros. This procedure does not cause the Extended Dependence variable to become significant either. In constructing Model 8, Chen’s study replaces the Democratic Dyad variable that is operationalized through the Unified Democracy Scores (UDS) with a new Democratic Dyad measure gathered from the Polity dataset. When Chen’s Model 8 is re-assessed using interstate war, I find the Extended Dependence variable significant at the 0.05 level (the *p*-value is 0.04). It appears that extended trade ties through defense pacts reduce the risk of interstate war, although their effect is trivial. Finally, Model 9 adds directed-dyad fixed-effects when the dependent variable is interstate war. The effect of Extended Dependence does not come forward as Chen’s study claims.

To save space, I relegate substantive tests of Extended Dependence to S3 Appendix.

## Heckman selection model of extended dependence and international conflict initiation

The replication analysis shows an insignificant relationship between extended dependence and interstate war. This null finding may result from the rarity of interstate war in the sample data. Although Chen and I use rare event logit–the standard statistical model designed to correct for the biases in logistic regression that occur when predicting rare events [34], I decide to explore the rarity of events further. When Chen [13 page 253] claims that “defensive alliance obligations are clearer when military conflict is more severe and intense,” he presumes that dyadic states have already become involved in conflict. If this presumption is correct, it needs to be empirically tested, which Chen overlooked in his article. By considering conflict dyads at levels below interstate war, I isolate the effect of extended dependence on interstate war. I employ a Heckman selection model for predicting conflict in the first stage and then move on to analyzing the effect of extended dependence on interstate war in the second stage, where the latter includes only conflict dyads. For the same reason, various existing studies adopt a Heckman selection model [e.g., 35].

Table 2 shows the results of the Heckman selection models. For identification purposes, I exclude Mutual Defensive Allies Count and Dyadic Defense Pact from the fatal MID model because the selection equation (Model 1) should contain at least one variable that is not in the outcome equation (Model 2). Those two variables should be closely related to the onset of militarized disputes, as hypothesized by Chen. However, once a dispute has been initiated, regardless of alliance ties, those two variables become less relevant to the onset of war since states are already aware of the presence of a contentious issue between them.

**Table 2:** Heckman selection model of extended dependence and international conflict initiation.

	DV: All MIDs	DV: Fatal MIDs	DV: All MIDs	DV: War
	1st Stage	2nd Stage	1st Stage	2n Stage
	Model 1	Model 2	Model 3	Model 4
Extended Dependence	-0.004**	-0.009*	-0.004**	-0.013
	(0.001)	(0.004)	(0.001)	(0.010)
Dyadic Dependence	-0.002	0.007	-0.002	-0.096
	(0.003)	(0.010)	(0.003)	(0.058)
Challenger Total Capability Share	0.302***	0.007	0.302***	-0.233
	(0.047)	(0.170)	(0.047)	(0.323)
Mutual Defensive Allies Count	-0.000		-0.000	
	(0.004)		(0.004)	
Dyadic Defense Pact	-0.026		-0.024	
	(0.083)		(0.084)	
Target Major Power	0.571***	0.153	0.570***	-0.435
	(0.063)	(0.177)	(0.063)	(0.464)
Challenger Democracy	-0.075***	-0.038	-0.075***	0.168
	(0.022)	(0.050)	(0.022)	(0.108)
Target Democracy	-0.046	-0.061	-0.046	0.006
	(0.025)	(0.058)	(0.025)	(0.121)
Contiguity	1.211***	0.354	1.210***	0.128
	(0.054)	(0.265)	(0.054)	(0.527)
Distance	-0.164***	-0.149*	-0.164***	0.011
	(0.020)	(0.061)	(0.020)	(0.127)
Year Fixed Effects	Yes		Yes	
Temporal Dependence	Yes		Yes	
Observations	1,362,097		1,362,097	

*Note*: Heckman’s probit is used for estimation. Entries in parentheses are Huber standard errors clustered on the dyad.

Robust standard errors in parentheses.

*p < .05

**p < .01

***p < .001, two-tailed tests.

Models 1 and 2 are the first set of selection models. The first stage tests for the influence of extended dependence in discouraging the initiation of any MID, and the second stage examines whether MIDs that have occurred will escalate into fatal MIDs. These two models are constructed to test Chen’s implicit theoretical expectation. The pacifying effect of Extended Dependence appears to emerge after I estimate the first and second stages simultaneously. However, the simultaneous estimation does not necessarily make the impact of Extended Dependence on fatal MIDs stronger in the second stage since it is significant only at the 0.05 level.

Models 3 and 4 are the second set of selection models in which the effect of extended dependence on any MID is examined in the first stage and interstate war in the second stage, predicted only if a MID equals 1. This simultaneous estimation ensures that any correlation between the initiation of a MID and its escalation to war is adequately considered. As I argue, the Extended Dependence variable does not engender a peace-building effect as it fails to achieve significance. This null result does not deviate from that in Table 1 and S2 Appendix.

## Reverse causality

To avoid reverse causality, Chen [13 page 251] lags all the predictors one year behind the outcome variable. I follow Chen’s approach since lagging is the most common practice among political scientists. Even though Chen and I account for potential reverse causality through lagging, I decide to go one step further by introducing an advanced estimation method. Following Roodman [36], I employ Arellano–Bond panel-data estimation [37] with a one-step difference generalized method of moments (GMM). The GMM model effectively deals with the problem caused by endogenous explanatory variables by utilizing their lagged levels as instruments for the difference equation and their lagged differences as instruments for the level equations. Numerous studies rely on the GMM model to account for endogeneity bias [e.g., 38]. I treat Extended Dependence and war as endogenous, but all other regressors in the model as exogenous. I report the GMM results in Model 1 in Table 3, which are consistent with those in the main analysis. Extended Dependence is not a significant predictor of interstate war, even after considering the reverse causality issue.

**Table 3:** Extended dependence and international conflict initiation: Reverse causality.

	DV: War
	GMM Model 1
Extended Dependence_*t-1*_	5.91e-06
	(0.000)
Dyadic Dependence_*t-1*_	-0.000
	(0.000)
Challenger Total Capability Share_*t-1*_	0.010
	(0.006)
Mutual Defensive Allies Count_*t-1*_	0.000
	(0.000)
Dyadic Defense Pact_*t-1*_	-0.018
	(0.012)
Target Major Power_*t-1*_	0.011
	(0.007)
Challenger Democracy_*t-1*_	-0.000
	(0.001)
Target Democracy_*t-1*_	-0.005
	(0.003)
Contiguity_*t-1*_	0.084*
	(0.039)
Distance_*t-1*_	-0.003
	(0.002)
War_*t-1*_	-0.056
	(0.122)
War_*t-2*_	-120.752*
	(55.746)
Year Fixed Effects	Yes
Temporal Dependence	Yes
Observations	1,270,619

*Note*:

*p < .05

**p < .01

***p < .001, two-tailed tests.

## Concluding remarks

By proposing a novel theory–Extended Dependence, Chen’s study significantly contributes to the literature on liberal peace. Drawing on a battery of empirical models and a series of robustness checks, the study demonstrates the pacifying effect of Extended Dependence on all and fatal militarized disputes. However, the study overlooks whether Extended Dependence reduces the likelihood of interstate war. My reexamination builds on the Extended Dependence Theory and applies it to the highest–and most pertinent–level of conflict. My reexamination is performed in the spirit of Chen’s [13 page 253] insights: “defensive alliance obligations are clearer when military conflict is more severe and intense, where Extended Dependence can thus produce an even stronger effect on reducing the incidence of military conflict initiation” [for replication examples, see 16, 39–43]. To my surprise, my reexamination fails to produce supporting evidence for Chen’s claim. As noted earlier, extended dependence appears to have a ceiling effect. In theory, Extended Dependence should dampen the likelihood of war; in reality, it does not exert a significant pacifying effect on war. This null finding suggests that the military allies of the target state may not be a critical factor in shaping the conflict behavior of challenging states. The Extended Dependence Theory fails to explain how economic linkages and security institutions work together to reduce the risk of the most destructive conflict–interstate war. To move the field of international relations forward, future scholars should further develop the theory of Extended Dependence since liberal peace is about how to reduce interstate *war* between trading partners. Preventing trivial conflicts such as verbal threats and accidental vessel collisions is insufficient for its aspirations.

## Supporting information

S1 Appendix U.S.-Japan trade. (DOC)

S2 Appendix Robustness tests. (DOC)

S3 Appendix Average marginal effects. (DOC)
