# Supplementary material for: Can trade and security alliance help reduce interstate war?
Source: PLoS One. 2024 Jun 20;19(6):e0304482. doi: 10.1371/journal.pone.0304482 (PMC11189244; doi:10.1371/journal.pone.0304482)
Supplement: S1 Appendix — (DOC) [file pone.0304482.s001.doc]

The United States and Japan are among the world’s largest economic powers. Appendix Figure 1 displays their trade for the years 1952-2014. The data comes from the Correlated of War trade project (version 4.0). Despite territorial disputes over the Tokdo Islands, the U.S., a key military ally of South Korea (the target), has not reduced its trade with Japan (the challenger). Figure 1 indeed indicates that the U.S.-Japan trade has been on the rise during the past six decades. Some decreases in the 2000s were due mainly to economic problems, such as the Japanese Import Restrictions on U.S. Beef in 2003 and the global financial crisis that occurred between mid-2007 and early 2009 (see Cooper 2014).

Cooper, William. 2014. “U.S.-Japan Economic Relations.” Congressional Research Service. RL32649.
