# Supplementary material for: Can trade and security alliance help reduce interstate war?
Source: PLoS One. 2024 Jun 20;19(6):e0304482. doi: 10.1371/journal.pone.0304482 (PMC11189244; doi:10.1371/journal.pone.0304482)
Supplement: Supplementary file 2 [file pone.0304482.s002.doc]

S2 Appendix Table 1 Extended Dependence and International Conflict Initiation: Robustness Checks

	DV: Wars								
	Politically Relevant Dyads	Sum Measure	Specific Alliances		More Controls	Outliers dropped	Listwise Deletion	Democratic Dyad	Directed-Dyad FE
Variable	Model 1	Model 2	Model 3	Model 4	Model 5	Model 6	Model 7	Model 8	Model 9
Extended Dependence	-0.063		-0.069	-0.079	-0.061	-0.072	-0.048	-0.098*	-0.193
	(0.046)		(0.044)	(0.046)	(0.039)	(0.045)	(0.036)	(0.048)	(0.115)
Dyadic Dependence	-0.717		-0.551	-0.556	-0.393	-0.547		-0.657	-0.697
	(0.571)		(0.434)	(0.430)	(0.368)	(0.430)		(0.467)	(0.460)
Chal. Total Cap. Share	-0.124	0.817*	0.811*	0.833*	0.639	0.830*	1.167**	0.707*	1.915
	(0.503)	(0.371)	(0.377)	(0.369)	(0.414)	(0.369)	(0.448)	(0.354)	(1.058)
Mu. Defen. Allies Count	-0.024	0.028	0.014	0.013	0.057	0.012	-0.008	0.026	0.093
	(0.042)	(0.040)	(0.041)	(0.041)	(0.041)	(0.039)	(0.039)	(0.042)	(0.118)
Dyadic Defense Pact	0.102	-0.126	-0.008	-0.024	0.213	0.007	0.831	-0.072	0.330
	(0.699)	(0.699)	(0.695)	(0.706)	(0.639)	(0.687)	(0.693)	(0.694)	(1.182)
Target Major Power	0.266	1.637***	1.706***	1.676***	1.526**	1.720***	2.156***	1.757***	n/a
	(0.495)	(0.444)	(0.494)	(0.488)	(0.466)	(0.490)	(0.473)	(0.488)	
Challenger Democracy	0.120	0.234	0.209	0.206	0.129	0.209	0.252		-0.954*
	(0.157)	(0.140)	(0.138)	(0.136)	(0.141)	(0.137)	(0.164)		(0.417)
Target Democracy	-0.602	-0.848**	-0.826*	-0.881**	-0.767**	-0.842**	-1.134**		-0.897
	(0.337)	(0.309)	(0.335)	(0.326)	(0.282)	(0.309)	(0.396)		(0.462)
Contiguity	0.989*	2.602***	2.640***	2.635***	2.585***	2.630***	2.356***	2.723***	0.551
	(0.428)	(0.408)	(0.400)	(0.399)	(0.415)	(0.400)	(0.428)	(0.398)	(0.819)
Distance	-0.308*	-0.460***	-0.480***	-0.485***	-0.638***	-0.487***	-0.277	-0.469***	-1907444.674
	(0.137)	(0.132)	(0.127)	(0.128)	(0.144)	(0.128)	(0.159)	(0.126)	(6237391.804)
Extended Depen. + Dyadic Depen.		-0.109							
		(0.061)							
Target U.S. Defense Pact			-0.082						
			(0.699)						
Target NATO member				0.745					
				(0.882)					
Target Total Defen. Allies Count					-0.035				
					(0.027)				
Alliance Portfolio Similarity					-2.543**				
					(0.969)				
Co-IGO Memberships					-0.027				
					(0.020)				
Dyadic Dependence after listwise deletion							-0.406		
							(0.338)		
Democratic Dyad								-1.671*	
								(0.766)	
Directed-Dyad Fixed Effects									Yes
Year Fixed Effects	Yes	Yes	Yes	Yes	Yes	Yes	Yes	Yes	Yes
Temporal Dependence	Yes	Yes	Yes	Yes	Yes	Yes	Yes	Yes	Yes
Observations	142,049	1,362,097	1,362,097	1,362,097	1,361,306	1,361,223	1,013,749	1,362,097	3,498

Notes : Robust standard errors are in parentheses. *p <0.05; **p <0.01; ***p <0.001, two-tailed tests.
