# Supplementary material for: Can trade and security alliance help reduce interstate war?
Source: PLoS One. 2024 Jun 20;19(6):e0304482. doi: 10.1371/journal.pone.0304482 (PMC11189244; doi:10.1371/journal.pone.0304482)

S3 Appendix Fig 2. Average Marginal Effects

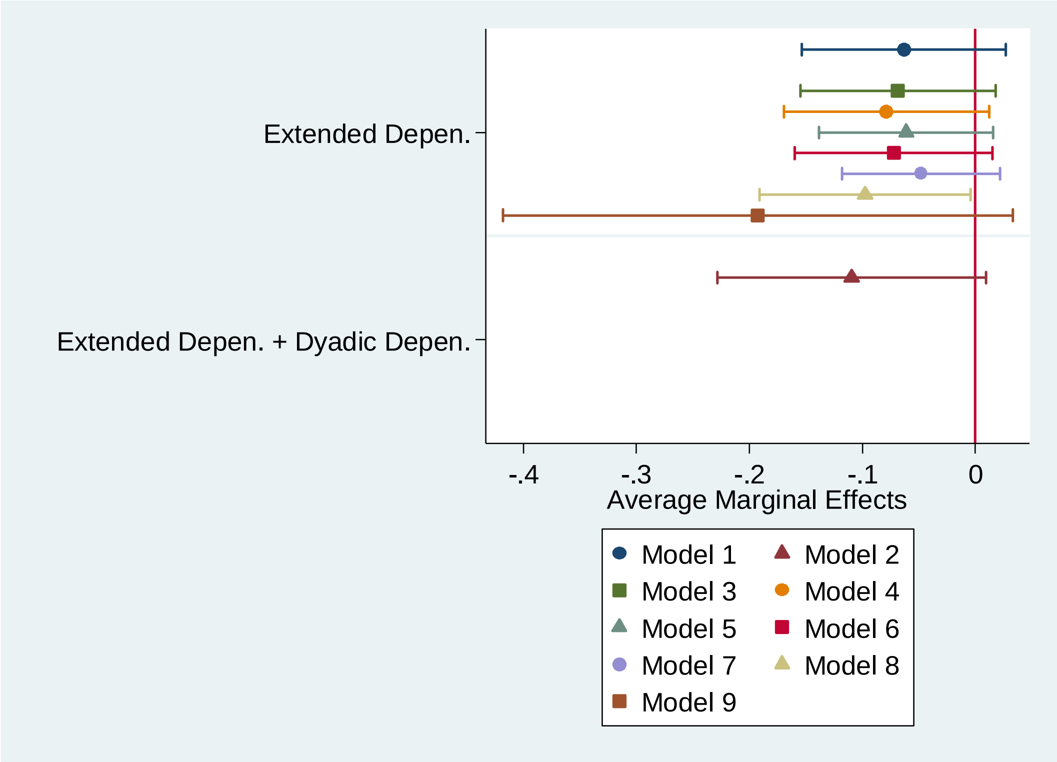

Supplement: Supplementary file 3 [file pone.0304482.s003.doc]
